# Acute Salicylate Toxicity: A Narrative Review for Emergency Clinicians

**DOI:** 10.7759/cureus.91505

**Published:** 2025-09-02

**Authors:** Alexander M Sidlak, Anthony Spadaro, Alex Koyfman, Brit Long

**Affiliations:** 1 Emergency Medicine, George Washington University, Washington, DC, USA; 2 Emergency Medicine, Rutgers University Hospital-Newark, Newark, USA; 3 Emergency Medicine, University of Texas Southwestern Medical Center, Dallas, USA; 4 Emergency Medicine, University of Virginia School of Medicine, Charlottesville, USA

**Keywords:** alkalinization, aspirin, hemodialysis, renal, salicylates, toxicology

## Abstract

Acute salicylate toxicity is a major source of morbidity and mortality. A multitude of medications, including over-the-counter therapies and topical treatments, contain salicylates. In overdose, they result in uncoupling of oxidative phosphorylation, inhibition of citric acid dehydrogenases, increased keto-acid production, and decreased ATP production, leading to a progression of signs and symptoms. Patients may develop respiratory alkalosis and an anion gap metabolic acidosis. Patients present with a range of symptoms, including nausea, vomiting, diarrhea, tinnitus, altered mental status, agitation, seizures, pulmonary edema, and coma. Acute treatment includes gastrointestinal decontamination, serum alkalinization, and fluid resuscitation. Hemodialysis is necessary in cases with severely high serum salicylate concentrations, hypoxia, altered mental status, and significant acid-base disorders. This review highlights the pearls and pitfalls of acute salicylate toxicity, including presentation, diagnosis, and management in the emergency department based on current evidence.

## Introduction and background

Salicylate toxicity results from an excess ingestion or the dermal absorption of salicylate-containing medications or topical products [1-3]. A therapeutic single dose of acetylsalicylate (aspirin), the most common source of salicylates, is 10 mg/kg with a daily range of 40-60 mg/kg/day. There are many other over-the-counter products used for their anti-inflammatory and analgesic properties that contain salicylates, and overdoses of any of them can result in toxicity [3,4]. All forms of salicylate (e.g., methyl salicylate, bismuth subsalicylate, acetylsalicylate, etc.) are metabolized to salicylate in the body, though a standard dose may have variable amounts of salicylate depending on the product [1,5]. Salicylate toxicity causes a myriad of metabolic, neurologic, and organ-specific effects, including tinnitus, encephalopathy, seizures, and pulmonary edema. Acute exposures are defined as a dose of 150 mg/kg over a period of fewer than eight hours [1].

## Review

Methodology

This narrative review provides a focused overview of acute salicylate toxicity for emergency clinicians. The goal of the review was to summarize the pathophysiology, emergency department (ED) presentation, and ED management of acute salicylate toxicity while addressing common pitfalls in the condition’s evaluation and management. To construct this narrative review, we searched PubMed and Google Scholar for English-language articles from database inception to January 10, 2025, using the keyword and Medical Subject Headings “salicylate” OR “aspirin” AND “poisoning” OR “toxicity” OR “overdose” OR “adverse effects.” The initial literature search revealed 98 full-text articles. Article inclusion was determined by author review and consensus based on clinical relevance to ED evaluation and management. We selected these articles through a consensus between two authors (AS and TS), with any disagreements resolved by discussion with a third author (BL). When available, retrospective and prospective studies, systematic reviews/meta-analyses, and clinical guidelines were preferentially selected over case reports that provided similar, but less detailed information. Articles discussing “chronic toxicity” as opposed to acute toxicity were excluded. We included a total of 55 resources for use in the narrative review based on relevance to emergency clinicians, supplemented by additional sources, including textbook chapters on salicylate testing. Using the selected articles, this narrative review discusses relevant pathophysiology, patient presentation, evaluation of suspected salicylate toxicity, and management.

Mechanism of toxicity

Salicylates cause a multitude of metabolic derangements, most notably metabolic acidosis, ketosis, and hypoglycemia (with initial hyperglycemia), which are ultimately related to an uncoupling of oxidative phosphorylation, inhibition of citric acid dehydrogenases, and decreased ATP production [2,5,6]. Lactate acid concentrations are often normal early in salicylate toxicity, and even later elevations in lactate or rising salicylate concentrations do not fully explain the metabolic acidosis [5]. The primary driver is the uncoupling of oxidative phosphorylation, which disrupts glucose metabolism and impairs cellular energy production. This process leads to excessive proton generation through ATP dissociation to ADP, a mechanism distinct from lactate production, and contributes directly to acidemia [5]. Salicylates also irreversibly inhibit cyclooxygenase, which contributes to ototoxicity, gastric irritation, and impaired platelet aggregation [2].

The pathophysiology and natural progression of toxicity are related to salicylic acid’s pharmacokinetics. In an acidic environment (e.g., the stomach), a larger percentage of salicylate exists as salicylic acid, a weak acid with a pKa of 2.97, which is the protonated and uncharged form of the molecule. As pH drops from 7.4 to 7.1, the proportion of uncharged salicylate more than doubles. The uncharged salicylic acid is more easily absorbed from the gastrointestinal (GI) tract and more readily crosses plasma membranes [5]. Once absorbed, the volume of distribution varies with changes in serum pH. With acidemia, a larger proportion of the molecule exists as the uncharged form that crosses into tissues, and the volume of distribution increases. As the volume of distribution increases, the toxic effects of salicylates increase as more salicylate is present in tissues. Hypoalbuminemia and chronic use of salicylates can also increase the volume of distribution and thus the tissue stores of salicylates (total body burden). Therefore, toxicity can occur at lower serum concentrations as it does in “chronic toxicity” or with decreasing serum concentrations as salicylate moves from serum to tissue in the midst of worsening acidemia [5,7,8]. After absorption, salicylates are metabolized via glucuronidation, oxidation, and glycine conjugation to other metabolites that have lesser effects in the body [8]. As salicylate concentrations increase, enzymatic activity becomes saturated, and metabolism of salicylates shifts from first to zero kinetics, where a smaller fixed amount becomes excreted per unit time, often increasing the observed half-life and duration of toxicity [5].

Tinnitus can occur at both therapeutic doses and with mild toxicity at concentrations approximating 30 mg/dL (just above the reported therapeutic range of 15-30 mg/dL). Though often defined as a ringing sensation, in acute salicylism, this largely manifests as a diminished ability to hear [9]. Respiratory alkalosis may also develop early on in toxicity. The alkalosis is caused by direct effects of salicylates on both the brainstem and arterial chemoreceptors, resulting in tachypnea and corresponding increases in tidal volume [10-12]. As toxicity progresses, hypokalemia and metabolic acidosis occur, allowing for greater entry of salicylate into the central nervous system (CNS) and other cells. Patients can then experience encephalopathy and direct myocardial effects due to the uncoupling of oxidative phosphorylation and decreased energy production in both the CNS and myocardium [5]. Patients can deteriorate rapidly as worsening toxicity and its manifestations (seizures, obtundation) further lower serum pH, leading to increased tissue concentrations and thus worsening toxicity [5].

Health system impact

With the availability of over-the-counter salicylate-containing products and the prevalence of aspirin use in adults (approximately one-third of adults use a daily aspirin for atherosclerotic cardiovascular disease prevention), salicylate toxicity presents an ongoing risk of mortality and morbidity with both acute overdoses and chronic toxicity [1,13]. In a review of approximately 14,000 admissions, more than 20% of patients developed failure of at least one organ system. In-hospital mortality in this study was 1% [14]. Risk factors associated with mortality were older age (>30 years), Asian/Pacific Islander race, diabetes mellitus, hyponatremia, ventricular arrhythmias, and organ failure (kidney, respiratory, and/or neurologic) [14]. Renal failure is the most common organ dysfunction reported [14]. In 2022, salicylate toxicity was one of the 25 most common causes of mortality reported to poison centers, representing 2.2% of all single-substance reported fatalities [4]. In pediatric patients, the frequency of salicylate toxicity and mortality has declined from its peak in the 1950s-1970s, when it was the most frequent cause of death in children. More recent data suggest it is rarely the cause of pediatric mortality, with zero pediatric deaths in 2004 from salicylate toxicity [1]. Currently, salicylate toxicity represents 0.013-0.017% of all pediatric and adult hospital admissions [14].

Sources of salicylates

Toxicity can arise from any of the various forms of salicylate. Many forms are available over the counter, and overdoses occur on more than just acetylsalicylate (aspirin), which itself exists in multiple dosages (Table 1). Over-the-counter formulations of aspirin for analgesia may contain as much as 500 mg/dose and may inadvertently be used concurrently with aspirin for secondary prevention of atherosclerotic disease, increasing the risk of toxicity. Additional sources of salicylates include bismuth subsalicylate (Pepto Bismol™), methylsalicylate (oil of wintergreen), and acetylsalicylate found in analgesic combination products such as Excedrin™ (aspirin, acetaminophen, caffeine) and Fiorinal (aspirin, butalbital, caffeine) [4,15-17]. Topical products are rarely associated with severe toxicity and mortality. If excessive amounts are used, especially on skin with a breakdown of the epidermal barrier due to disease (e.g., psoriasis), toxicity can result [18]. Dermal toxicity has also been reported with the use of artificial barriers (i.e., plastic wrap) even with intact skin [18]. Methyl salicylate is commonly found in multiple over-the-counter topical analgesics [3]. Ingestions of methyl salicylate in the form of oil of wintergreen, a flavoring agent used in baking, can also occur, and given the high concentration of salicylate and sweet taste, are especially dangerous to pediatric patients, as they can easily ingest a toxic dose. A single tablespoon of oil of wintergreen contains enough methyl salicylate to produce salicylate toxicity in pediatric patients [1,3,17,19].

**Table 1:** Substances with salicylates.

Substances
Alka-Seltzer
Aspirin (81 mg chewable, 325 mg enteric-coated, 500 mg Bayer Back and Body)
Bismuth subsalicylate (e.g., Pepto Bismol, Maalox Total Relief)
Oil of wintergreen
Over-the-counter cough, cold, and flu medications
Over-the-counter migraine and headache medications (e.g., Excedrin, BC powder, Goody’s powder)
Topical analgesics (e.g., Bengay, Icy Hot, Aspercreme)
Wart removers

Clinical presentation of toxicity

The most common presentations of salicylate toxicity include intentional overdose or inadvertent overdose in patients who chronically utilize aspirin-containing products. After an acute ingestion, patients may present at variable times after ingestion and thus manifest different symptoms depending on the degree of toxicity and time after ingestion. Some may present early, simply reporting an ingestion, and may be initially asymptomatic. Aspirin, methyl salicylate, and keratolytic salicylic agents are irritating and potentially corrosive to the GI tract. Nausea, vomiting, and abdominal pain are common early (<6 hours) symptoms after a large ingestion [1,20]. Toxicity may develop at variable times after an acute ingestion, with the majority of patients developing toxicity 4-12 hours post-ingestion [1]. Tinnitus will also occur as concentrations increase, but it is not found in all patients; in one study, 78% of patients had tinnitus [9]. The average concentration at which tinnitus occurs approximates 30 mg/dL, but it can start at concentrations as low as 19.6 mg/dL [9]. Historically, before the possibility of measuring salicylate concentrations, aspirin used for rheumatologic conditions would be uptitrated until tinnitus developed [9].

Most challenging are cases without a clear exposure history and undifferentiated patients with altered mental status. With late-presenting aspirin toxicity, non-cardiogenic pulmonary edema, encephalopathy, and seizures may occur. Dysrhythmias ranging from ventricular tachycardia to complete heart block have also been documented in case reports of severe toxicity [21,22]. Cardiac effects are likely dose-dependent and reported in near-death patients [5,21,22]. As toxicity worsens, patients may develop altered mental status and become comatose. In one series of salicylate fatalities, 31.5% of patients who later died were comatose on arrival to the hospital [23]. Hyperthermia (>40°C) is uncommon after most acute overdoses. It has been reported in cases where death has occurred despite treatment and likely represents a preterminal manifestation of severe toxicity [5,6,24,25].

ED evaluation

Patients with suspected salicylate toxicity require a focused history, examination, and laboratory analysis in the ED. With any overdose, testing for salicylates is reasonable, especially as analgesics and cough medications are often coformulated with salicylate products. Patients may additionally misidentify the over-the-counter analgesic they ingested. Nevertheless, in most scenarios, the identification of an elevated salicylate concentration after an unknown overdose or in patients with no report of salicylate ingestion is uncommon [26]. However, some presentations have an increased odds of salicylate toxicity, in which testing should be considered. The presence of altered mental status or metabolic acidosis makes salicylate toxicity more likely, even in the absence of a reported ingestion [27]. Especially in patients who present after an overdose with no corroborating history, salicylate testing should be performed. Additionally, patients with salicylate toxicity often have respiratory alkalosis, and testing should be considered in patients with tachypnea or a respiratory alkalosis that is not otherwise explained [2]. Pediatric topical exposure to methyl salicylate or exploratory ingestions of oil of wintergreen may cause salicylate toxicity, and a salicylate concentration should be obtained in patients with potential exposure to these compounds [1,3].

Respiratory alkalosis, defined as an increased minute ventilation relative to carbon dioxide production secondary to tachypnea and/or hyperpnea, can indicate salicylate toxicity [28,29]. Bedside evaluation is important in identifying these physiologic changes, as the recorded respiratory rate is often inaccurate: they may have recorded a rate that is erroneously normal or may have a truly normal rate but be hyperpneic and have increased tidal volume breathing [30]. Co-ingestions often obscure some of the signs, symptoms, and laboratory findings classically related to aspirin toxicity. With sedatives, especially, both respiratory alkalosis and tinnitus may be absent. Sedatives and analgesic agents, including opioids, can blunt the compensatory respiratory alkalosis that develops and, by doing so, decrease the pH, increase CNS penetration, and worsen toxicity. In one small case series, only one-fourth of patients had respiratory alkalosis; however, 33% of patients had co-ingestions of other drugs, and those with co-ingestions had a lower incidence of respiratory alkalosis [10].

With acute overdoses reported to family, pre-hospital personnel, or in the ED, the need for testing and potential management of salicylate toxicity is clear. Most often, patients will report a history of salicylate ingestion, often in a suicide attempt, and emergency clinicians should ask about the intent behind an ingestion. The dose, formulation, and time of ingestion should be obtained. The clinician should also evaluate the presence of co-ingestants based on history, presence of toxidromes, and laboratory abnormalities. Occasionally, the analgesic taken is misreported, and a salicylate and acetaminophen concentration should be obtained in any reported suicide attempt.

All patients with suspected, reported, or confirmed salicylate toxicity should have a laboratory evaluation that includes a venous or arterial blood gas, complete blood count, electrolytes, renal and liver function, serum acetaminophen and salicylate concentrations, and an electrocardiogram. The measurement of the acid-base status and the degree of acidemia is important in assessing the degree of toxicity. As discussed previously, concentrations are important in the context of the acid-base status of the patient, as this determines the amount of uncharged and free salicylate, the form of the molecule that crosses tissues and leads to toxicity. In a prospective cohort study designed to identify early predictors of severe in-hospital outcomes defined as acidosis (pH <7.3 or bicarbonate <16), hemodialysis (HD), or death, studies found that age, respiratory rate, lactate concentration, coma, and co-ingestions were associated with severe outcomes [31]. When adjusted for initial salicylate concentrations, age (odds ratio = 1.13) and respiratory rate (odds ratio = 1.29) were independent predictors of severe outcomes [31]. These factors can assist with early prognostication in salicylate toxicity.

A chest X-ray may be useful to evaluate for alternative etiologies of respiratory abnormalities or salicylate-induced pulmonary edema [32]. For patients with undifferentiated altered mental status and no corroborating information, other testing, including CT, blood cultures, and lumbar puncture, may be obtained depending on the clinical scenario. In some cases, salicylate toxicity may not even be considered until common alternative etiologies have been excluded. The typical and subtle findings discussed previously in this section can help make the diagnosis of salicylate toxicity earlier in the undifferentiated altered patient.

Classically, salicylate toxicity produces a mixed anion gap metabolic acidosis with respiratory alkalosis, but there are nuances to laboratory assessment in salicylate toxicity. Occasionally, the anion gap may be normal. This is due to the interference of salicylate with some analyzers of chloride, which leads to a falsely elevated chloride and a falsely decreased or normal anion gap [33-35]. In one study where the center used the Siemens Dimension Vista analyzer (Newark, DE), a 15% false increase in chloride was present when salicylate concentrations were greater than 60 mg/dL [34]. Hypokalemia may be present and, as discussed below, has implications for the treatment of salicylate toxicity. Mixed acid-base disorders and concomitant metabolic alkalosis from vomiting may also lead to an apparently normal anion gap [10]. Because the metabolic acidosis is secondary to uncoupling of oxidative phosphorylation, lactic acid may not become elevated initially as energy production is not shunted toward anaerobic metabolism [5]. However, elevations in lactic acid on presentation are associated with severe outcomes, with a lactate >2.25 mmol/L being 78% sensitive for severe outcomes [31].

In a review of hospitalizations for salicylate toxicity using a national sample, the most common laboratory abnormalities were hypokalemia (25.4%), anion gap metabolic acidosis (19.1%), and alkalosis (11.1%) [13]. Infrequently encountered overdoses on uncouplers of oxidative phosphorylation, including dinitrophenol, chlorophenoxy herbicides, and arsenate (As5+), may lead to a similar metabolic acidosis [24,25]. Other conditions causing acidosis may be on the differential and include starvation ketosis, alcoholic ketoacidosis, and diabetic ketoacidosis [2,5].

Glucose abnormalities are often present in salicylate toxicity, both in the serum and cerebrospinal fluid. Hyperglycemia may be present early in toxicity, occasionally reaching concentrations of 200-300 mg/dL as the body attempts to compensate for impaired ATP production with increased epinephrine release [5]. Later, hypoglycemia is common as gluconeogenesis is inhibited, and cases of refractory hypoglycemia have been reported [36,37]. Even if serum glucose is normal, CNS glucose may be low or lower than the typical two-thirds ratio to serum glucose (hypoglycorrhachia), and this may contribute to encephalopathy [38].

In terms of testing directly for salicylates, a single test is often insufficient, and patients with acute toxicity require serial concentrations to determine if they will develop toxicity and to determine how significant the overdose will be [39-43]. Unfortunately, even a serum salicylate concentration may be misleading as to the degree of toxicity and the ultimate prognosis of salicylate toxicity. Though a nomogram can be used to predict potential toxicity from other overdoses, such as acetaminophen, no reliable one exists for salicylates [39]. Because of erratic kinetics and delayed toxicity, if patients present shortly after ingestion, it may be important to check multiple salicylate concentrations serially to exclude the potential for toxicity [40,41]. In one study where patients who developed toxicity and needed treatment with sodium bicarbonate, an initial serum salicylate concentration was undetectable up to four hours after ingestion [41]. Not only can salicylate concentrations be undetectable shortly after ingestion, but delayed peaks in serum salicylate can occur. In a study evaluating characteristics associated with severe toxicity, initial serum salicylate concentration had no association with outcome [31]. The potential for delayed toxicity occurs for several reasons, including pylorospasm and delayed gastric emptying, delayed absorption from enteric-coated tablets, and bezoar formation [2,5,42]. In one case report, salicylate toxicity did not occur until 35 hours after a single acute ingestion [43]. Continued dermal absorption from topical methyl salicylate has also been implicated in altered kinetics and rising salicylate concentrations despite enhanced elimination [21]. Therefore, in order not to miss the potential for toxicity after a reported salicylate ingestion, additional concentrations at two-hour intervals would need to be checked.

ED management

Consultation with a medical toxicologist or local poison control center is recommended in all patients with suspected or confirmed salicylate toxicity. All patients with suspected salicylate toxicity should be evaluated for the presence of ongoing exposure and have external decontamination if a topical form of salicylate exposure is present. Gastric decontamination should be utilized in those with acute toxicity if there are no contraindications (e.g., inability to protect the airway or refractory vomiting). Activated charcoal, 1 g/kg orally up to 100 g, can be effective in limiting absorption of salicylates by adsorbing the xenobiotic in the gut [1,44]. In a retrospective study on charcoal use, an admitting toxicology service in Australia used an initial 50 g dose and found a reduction in peak salicylate concentration compared to patients not given charcoal [44]. While charcoal is often administered as one dose, multiple doses may be administered if the patient can tolerate it (50 g every four hours for two more doses after the first or 25 g every two hours for three doses). Multi-dose activated charcoal given in this manner can reduce salicylate absorption if a bezoar has formed and reduce enterohepatic recirculation [5,45,46]. Correspondingly, even if given once, charcoal may be beneficial even later than the often-stated threshold of one to two hours after salicylate ingestion, given the delayed absorption, pylorospasm, and bezoar formation that can occur [1,5,42,44-46].

Patients are often hypovolemic due to vomiting and insensible fluid losses from hyperventilation and hyperthermia. Thus, intravenous (IV) fluid resuscitation is recommended to correct fluid losses, which can be done with any crystalloid, although, as discussed below, sodium bicarbonate-containing fluids are particularly useful. Expert sources have reported an initial fluid deficit of up to 4-6 L in patients with acute toxicity [5]. Sodium bicarbonate infusion is important not only to correct metabolic acidosis and improve urinary elimination of salicylate but also to alkalinize the serum and shift salicylate out of cells to minimize acute toxicity. Alkalinization with sodium bicarbonate will also prevent a spiral of worsening acidosis, higher tissue salicylate concentrations, and further deterioration. Sodium bicarbonate infusions should be started in all patients with metabolic acidosis (pH < 7.4), high serum salicylate concentrations (>30 mg/dL in acute toxicity), or with significant symptoms.

In salicylate toxicity, aggressive serum alkalinization is integral to management. This typically includes a bolus of 1-2 mEq/kg of hypertonic sodium bicarbonate followed by an infusion of isotonic sodium bicarbonate (150 mEq added to 1 L of 5% dextrose in water) infused at 2-3 cc/kg/hour, though there is no study to support specific dosing [2,5]. If toxicity is severe, additional boluses of hypertonic sodium bicarbonate may be required [5]. Increasing the serum pH to a goal of 7.50-7.55 will decrease the volume of distribution of salicylate: salicylate will shift out of the tissues and into the serum [5]. Urinary alkalinization aimed to achieve a urine pH >7.5 is a secondary goal, which shortens the duration of toxicity [44,46,47]. Maintaining normokalemia is important as urinary alkalinization cannot be achieved if hypokalemia is present [2]. Initial supplementation of potassium (40-60 mEq), addition of potassium (40-60 mEq) to the bicarbonate infusion, and additional administrations in response to monitoring of serum concentrations can be performed to maintain normokalemia [2,5].

Hypoglycemia and hypoglycorrhachia may contribute to altered mental status and should be corrected with IV dextrose [5]. For patients with initial mild encephalopathy, neuroglycopenia should be considered. This can occur even with normal serum or capillary glucose measurements. An IV dextrose bolus (0.5-1 g/kg) and/or infusion can mitigate or help resolve mild encephalopathy. Two cases of euglycemic patients with salicylate-induced delirium showed evidence of improvement after dextrose infusion [48]. Seizures should be managed with benzodiazepines, particularly in instances of polypharmacy, when the exact xenobiotic causing seizures may not be known [5]. Hypotension should be corrected with IV fluids and vasopressors as needed to prevent end-organ damage and worsening acidosis from poor perfusion.

Changes in mental status, including confusion, agitation, and coma, are suggestive of neurotoxicity and associated with significant morbidity and mortality in the setting of acute salicylate toxicity. HD is nearly always indicated in these cases and should be performed if severe neurotoxicity (coma or seizures) is present, regardless of serum concentrations, unless co-ingestions are contributing or hypoglycorrhachia is present [5,47,48,49]. Though any degree of encephalopathy should be concerning and raise suspicion for severe toxicity, if mental status improves with dextrose and fluids and salicylate concentrations are not significantly elevated, HD may ultimately not be required [47,48]. A review of fatalities identified through poison centers found a majority had peak salicylate concentrations less than 100 mg/dL, an oft-cited threshold for HD [50]. In another retrospective study including patients who were intubated for severe salicylate toxicity (>80 mg/dL), patients who did not receive HD all died, compared to survival in 83.3% who were dialyzed [50]. Therefore, reliance on concentration alone will lead to delays in treatment [39,51]. Clinical status, including whether a patient is encephalopathic and whether they can tolerate the serum and urinary alkalinization that must occur, is a more important factor in the consideration of HD [28,41,47,49]. The Extracorporeal Treatment in Poisoning Workgroup (EXTRIP) makes specific recommendations for when HD should be performed for salicylate toxicity [47]. HD is recommended in general for altered mental status, hypoxia, if standard therapies are not producing an adequate response, specific serum concentrations, or if pH is less than 7.20 (Table 2) [47]. HD should be considered based on clinical status and may need to be initiated if a patient has respiratory insufficiency or persistent encephalopathy. In the setting of comorbidities and non-cardiogenic pulmonary edema, a patient may not tolerate the fluids needed to achieve alkalinization, thus necessitating HD [47]. Intermittent HD due to the increased rate of clearance of salicylates is the preferred modality in acutely toxic patients [47]. HD should be continued until clinical improvement, and if concentrations are readily available, until salicylate concentrations decline below 19 mg/dL [47]. Although recommendations for these therapies are strong, the evidence supporting them is weak (level D) [47]. In one case report, a patient died despite two treatments with intermittent HD due to severe toxicity and persistently elevated concentrations [52]. Other therapies may be appropriate if HD cannot be performed or is unavailable. These include continuous renal replacement therapy [47,53].

**Table 2:** Dialysis indications for salicylate toxicity.

Indications for dialysis
Clinical situations for which dialysis is recommended
Altered mental status or confusion
Hypoxia requiring supplemental oxygen
Salicylate concentration >100 mg/dL or >90 mg/dL in the presence of impaired renal function
Failure of adequate response to standard supportive measures
Clinical situations for which dialysis is suggested
Salicylate concentration >90 mg/dL or >80 mg/dL in the presence of impaired renal function
If pH is less than 7.20

Clinicians must also consider co-ingestions. An altered patient with a low salicylate concentration may have developed encephalopathy secondary to another xenobiotic. Nevertheless, the altered mental status should not be discounted even though salicylate toxicity may not be the initial driver. The presence of sedative-hypnotic agents may cloud the clinical picture, limit compensation of metabolic acidosis, and increase the risk of decompensation if there is continued absorption of salicylate from the gut [5,10,42,43,45].

Intubation or any chemical restraint should be avoided, if possible, as sedation and paralysis may result in further decrease in pH, due to hypercarbia from apnea, shifting salicylate to tissues, which will further worsen salicylate toxicity and CNS and myocardial dysfunction. If necessary for safety or catheter placement, ketamine may be effective, although this has a risk of reducing tidal-volume ventilation and worsening acidosis. Non-invasive ventilation, such as a high-flow nasal cannula, may reduce the work of breathing.

Amid worsening toxicity or co-ingestions, intubation may become necessary despite its danger if patients are unable to protect their airway (e.g., recurrent vomiting or active seizures) or develop severe hypercapnia. If the patient requires endotracheal intubation, the clinician should attempt to mitigate the drop in serum pH that will occur from any apneic periods during an intubation [5,54,55]. There are no guidelines or randomized evidence to guide this process. Delayed sequence intubation after resuscitation and with agents that may not suppress the respiratory drive or awake-intubation should be considered to minimize apneic periods [55]. IV boluses of bicarbonate (2 mEq/kg bolus) 5-10 minutes before induction and paralysis, and hyperventilation with bag-valve mask ventilation should be considered to optimize acid-base status before intubation [55]. The most experienced clinician should perform the intubation to improve the chance of first-pass success and minimize the apneic time [55]. After intubation, the patient should be immediately connected to the ventilator, and the settings should be adjusted to ensure a higher minute ventilation with increased respiratory rate or tidal volume, targeting the patient’s respiratory rate and tidal volume before induction and paralysis. As the metabolic acidosis improves, the ventilator settings can then be adjusted [55].

Patients with severe toxicity will need laboratory monitoring every two to four hours and potentially require vasoactive medications and respiratory support, and thus admission to the intensive care unit setting is warranted. Patients with milder toxicity may potentially be managed on a medical-surgical floor [5,44]. Patients with slightly elevated salicylate concentrations without metabolic abnormalities may be observed in the ED for clinical stability and assurance of declining salicylate concentrations before obtaining psychiatric evaluation or discharge, depending on the clinical scenario [5,44]. Table 3 lists pearls in the evaluation and management of patients with acute salicylate toxicity.

**Table 3:** Pearls in salicylate toxicity.

Clinical presentation
Tinnitus, tachypnea, and altered mental status are signs and symptoms of salicylate toxicity
Hyperthermia developing after acute toxicity is uncommon and represents an end-stage finding identified mostly in cases where fatalities occur
Laboratory evaluation
Respiratory alkalosis and metabolic acidosis are characteristic laboratory findings in salicylate toxicity, but this is not always present
A single negative salicylate concentration too early after ingestion may not exclude salicylate toxicity
A low central nervous system glucose (hypoglycorrhachia), despite a normal or high serum glucose, contributes to altered mental status
Management
Gastrointestinal decontamination, serum alkalinization, and general supportive measures are the mainstay of emergent management
High serum salicylate concentrations, worsening confusion, pulmonary edema, or severe acid-base abnormalities may require hemodialysis
Intubation may be dangerous in salicylate toxicity, and, if necessary, pre-intubation conditions should be optimized. Mechanical ventilation post-intubation should target a high minute ventilation

Limitations

There are several limitations of this review. As this is not a systematic review but rather a narrative review, this manuscript did not follow the Preferred Reporting Items for Systematic reviews and Meta-Analyses guidelines and was not registered with PROSPERO, which may introduce bias in selecting more prominent articles or those that confirm the practices of the authors. However, authors with extensive experience with literature search and other narrative reviews completed the literature search and article selection, which should reduce this bias.

## Conclusions

Acute salicylate toxicity is a major source of morbidity and mortality. The presentation varies, including GI symptoms, tinnitus, altered mental status, seizures, and coma. Classic laboratory findings, such as respiratory alkalosis with an anion gap metabolic acidosis or even an elevated serum salicylate concentration, may not be present depending on the time course. GI decontamination, serum alkalinization, and fluid resuscitation are critical steps in the initial management of salicylate toxicity. Patients with encephalopathy, coma, high serum salicylate concentrations, hypoxia, and significant acid-base disorders require HD. Chemical sedation, physical restraint, and intubation are fraught with the potential to worsen toxicity by inducing apnea and reducing the necessary compensatory minute ventilation.
